# Supplementary material for: Diagnostic Utility of TSSC3 and RB1 Immunohistochemistry in Hydatidiform Mole
Source: Int J Mol Sci. 2023 Jun 2;24(11):9656. doi: 10.3390/ijms24119656 (PMC10253801; doi:10.3390/ijms24119656)
Supplement: Supplementary file 1 [file ijms-24-09656-s001.zip › ijms-2404562-supplementary.pdf]

Table S1: List of paternally-imprinted, maternally-expressed genes (Source: https://www.geneimprint.com/site/genes-by-species.Homo+sapiens.imprinted-M and https://www.proteinatlas.org/)

**Gene**	**Gene description**	**Aliases**	**Location**	**Protein expression and tissue localisation**
TP73	Tumour protein p73	P73	1p36.3	Nuclear expression in a subset of glandular cells in the fallopian tube, squamous epithelium and respiratory epithelium.
ADTRP	Androgen dependent TFPI regulating protein	AIG1L, C6orf105, dJ413H6.1	6p24.1 AS	NA
SLC22A2	Solute carrier family 22 member 2	OCT2, MGC32628	6q26 AS	Distinct membranous and cytoplasmic expression in renal tubules.
SLC22A3	Solute carrier family 22-member 3	EMT, EMTH, OCT3	6q26-q27	Cytoplasmic expression in most tissues.
HOXA4	Homeobox A4	HOX1, HOX1D	7p15-p14 AS	General nuclear expression.
MAGI2	Membrane associated guanylate kinase, WW and PDZ domain containing 2	AIP1, AIP-1, ARIP1, SSCAM, MAGI-2, ACVRIP1	7q21 AS	Cytoplasmic and nuclear expression in several tissues
PPP1R9A	Protein phosphatase 1 regulatory subunit 9A	NRB1, NRBI, FLJ20068, KIAA1222, Neurabin-I	7q21.3	Membranous and cytoplasmic expression in most tissues
DLX5	Distal-less homeobox 5	-	7q22 AS	Nuclear expression in most tissues
TFPI2	Tissue factor pathway inhibitor 2	PP5, REF1, TFPI-2, FLJ21164	7q22 AS	Selective cytoplasmic expression in placental trophoblasts.
CPA4	Carboxypeptidase A4	CPA3	7q32	NA
KLF14	Kruppel-like factor 14	BTEB5	7q32.3 AS	NA
SVOPL	SVOP like	-	7q34 AS	NA

KCNK9	Potassium two pore domain channel subfamily K member 9	KT3.2, TASK3, K2p9.1, TASK-3, MGC138268, MGC138270	8q24.3 AS	NA
OSBPL5	Oxysterol binding protein like 5	ORP5, OBPH1, FLJ42929	11p15.4 AS	General cytoplasmic expression with a granular pattern.
KCNQ1DN	KCNQ1 downstream neighbour	BWRT, HSA404617	11p15.4	NA
H19	H19 Imprinted Maternally Expressed Transcript	ASM, BWS, ASM1, MGC4485, PRO2605, D11S813E	11p15.5 AS	NA
SLC22A18	Solute carrier family 22-member 18	HET, ITM, BWR1A, IMPT1, TSSC5, ORCTL2, BWSCR1A, SLC22A1L, p45-BWR1A, DKFZp667A184	11p15.5	Luminal membranous expression in several tissues, most abundant in small intestines.
CDKN1C	Cyclin dependent kinase inhibitor 1C	BWS, WBS, p57, BWCR, KIP2	11p15.5 AS	Nuclear expression in several tissues.
PHLDA2	Pleckstrin homology like domain family A member 2	IPL, BRW1C, BWR1C, HLDA2, TSSC3	11p15.5 AS	NA
KCNQ1	Potassium voltage-gated channel subfamily Q member 1	LQT, RWS, WRS, LQT1, SQT2, ATFB1, ATFB3, JLNS1, KCNA8, KCNA9, Kv1.9, Kv7.1, KVLQT1, FLJ26167	11p15.5	Cytoplasmic and membranous expression mainly in adrenal gland, thyroid gland and stomach.
ANO1	Anoctamin 1	DOG1, TAOS2, ORAOV2, TMEM16A	11q13.3	Cytoplasmic and membranous expression in several tissues.
NTM	Neurotrimin	HNT, NTRI, IGLON2	11q25	High expression in CNS
RBP5	Retinol binding protein 5	CRBP3, CRBPIII, CRBP-III	12p13.31 AS	Cytoplasmic expression at variable levels in all tissues.
ATP5F1EP2	ATP Synthase F1 Subunit Epsilon	ATP5EP2	13q12.2	NA

	Pseudogene 2			
RB1	RB transcriptional corepressor 1	RB, pRb, OSRC, pp110, p105-Rb	13q14.2	Nuclear expression in most tissues, including retina.
SMOC1	SPARC related modular calcium binding 1	OAS	14q24.2	Cytoplasmic expression in testis.
MEG3	Maternally Expressed 3	GTL2, FP504, prebp1, PRO0518, PRO2160, FLJ31163, FLJ42589	14q32	NA
MEG8	Maternally Expressed 8	Bsr, Irm, Rian, SNHG23, SNHG24, lnc-MGC, LINC00024, NCRNA00024	14q32.2-q32.31	NA
DIO3OS	DIO3 Opposite Strand Upstream RNA	DIO3-OS, DIO3-AS1, C14orf134, NCRNA00041	14q32.31 AS	NA
SNORD113-1	small nucleolar RNA, C/D box 113-1	14q(I-1)	14q32.31	NA
SNORD114-1	small nucleolar RNA, C/D box 114-1	14q(II-1)	14q32.31	NA
UBE3A	Ubiquitin protein ligase E3A	AS, ANCR, E6-AP, HPVE6A, EPVE6AP, FLJ26981	15q11-q13 AS	Mainly cytoplasmic but also nuclear expression in all tissues.
ATP10A	ATPase phospholipid transporting 10A (putative)	ATPVA, ATPVC, ATP10C, KIAA0566	15q11.2 AS	NA
ZNF597	Zinc finger protein 597	-	16p13.3 AS	Low nuclear expression in a few tissues, most abundant in testis.
NAA60	NAA60	HAT4, NAT15	16p13.3	Cytoplasmic expression in most tissues.
TCEB3C	transcription elongation factor B polypeptide 3C	HsT829, TCEB3L2, Elongin A3	18q21.1 AS	NA
PARD6G	Par-6 family cell	PAR-6G,	18q23 AS	NA

	polarity regulator gamma	PAR6gamma		
NLRP2	NLR family pyrin domain containing 2	NBS1, PAN1, NALP2, PYPAF2, CLR19.9	19q13.42	NA

NA – Not available

Table S2: Expression of TSSC3 and RB1 in CT cells of CM, PM and NMA

No.	Sample No.	βhCG (mIU/mL)	Diagnosis	TSSC3		RB1	
				Intensity	%	Intensity	%
**1**	CM001	170,624	CM	-	0	1+	30
**2**	CM004	583,933	CM	1+	100	1+	30
**3**	CM005	170,000+	CM	-	0	1+	30
**4**	CM006	NA	CM	-	0	2+	20
**5**	CM007	67,899.50	CM	-	0	-	0
**6**	CM008	186,596	CM	-	0	2+	30
**7**	CM009	542,643	CM	-	0	2+	30
**8**	CM010	670,773.80	CM	-	0	2+	10
**9**	CM011	1,000,000	CM	1+	20	2+	40
**10**	CM012	200,382	CM	-	0	2+	30
**11**	CM013	25,000	CM	-	0	2+	30
**12**	CM014	34,985.90	CM	-	0	2+	33
**13**	CM016	187,479.50	CM	-	0	2+	20
**14**	CM017	NA	CM	1+	20	2+	20
**15**	CM018	NA	CM	-	0	1+	30
**16**	CM019	NA	CM	-	0	2+	100
**17**	CM020	NA	CM	-	0	2+	30
**18**	PM002	184,108.50	CM	3+	20	2+	50
**19**	PM003	181,113.20	CM	2+	50	2+	40
**20**	PM005	NA	CM	1+	10	2+	30
**21**	PM006	NA	CM	1+	10	1+	10
**22**	PM007	400,000	CM	1+	30	2+	20
**23**	PM011	NA	CM	-	0	2+	10
**24**	PM012	79,677	CM	-	0	2+	30
**25**	PM014	89,116	CM	2+	80	2+	10
**26**	PM015	131,942	CM	-	0	2+	40
**27**	PM016	79,284	CM	-	0	2+	30
**28**	PM021	NA	CM	-	0	2+	10
**29**	PM022	NA	CM	-	0	2+	60
**30**	PM008	9,515.40	PM	3+	30	2+	100
**31**	PM009	33,906	PM	3+	50	2+	100
**32**	PM010	NA	PM	2+	70	2+	100
**33**	PM017	NA	PM	2+	60	2+	100
**34**	PM018	NA	PM	3+	50	2+	100
**35**	PM020	11,764	PM	2+	60	2+	100
**36**	PM024	NA	PM	3+	20	2+	100
**37**	PM025	NA	PM	3+	20	2+	100
**38**	PM026	NA	PM	3+	30	2+	100
**39**	PM033	113,354	PM	2+	60	2+	100

**40**	PM036	369,109	PM	3+	10	2+	100
**41**	PM037	123,040	PM	3+	50	2+	100
**42**	PM038	321,637	PM	3+	50	2+	100
**43**	PM040	13,298	PM	2+	70	2+	100
**44**	POC011	19,132.60	PM	2+	60	2+	100
**45**	POC001	NA	NMA	3+	100	2+	100
**46**	POC002	NA	NMA	3+	80	2+	100
**47**	POC003	NA	NMA	3+	100	2+	100
**48**	POC004	NA	NMA	3+	100	2+	100
**49**	POC005	NA	NMA	3+	100	2+	100
**50**	POC006	NA	NMA	2+	50	2+	100
**51**	POC007	NA	NMA	3+	20	2+	100
**52**	POC008	NA	NMA	3+	30	1+	10
**53**	POC009	NA	NMA	3+	90	2+	100
**54**	POC010	NA	NMA	3+	90	2+	100
**55**	POC012	NA	NMA	2+	30	2+	100
**56**	CM002	14,604.40	NMA	1+	100	2+	100
**57**	PM013	NA	NMA	2+	80	2+	100
**58**	PM019	NA	NMA	3+	40	2+	100
**59**	PM023	< 1.2	NMA	3+	80	2+	100
**60**	PM027	25,888	NMA	3+	70	2+	100
**61**	PM028	< 1.2	NMA	3+	70	2+	100

CM – Complete mole, PM – Partial mole, NMA – Non-molar abortus, NA – Not available
